# Impact of Tigecycline Versus Other Antibiotics on the Fecal Metabolome and on Colonization Resistance to *Clostridium difficile* in Mice

**DOI:** 10.20411/pai.v2i1.159

**Published:** 2017-01-18

**Authors:** Robin L.P. Jump, David Kraft, Kelly Hurless, Alex Polinkovsky, Curtis J. Donskey

**Affiliations:** 1 Department of Medicine, Infectious Diseases Division, Case Western Reserve University School of Medicine, Cleveland, Ohio; 2 Geriatric Research, Education and Clinical Center, Cleveland Veterans Affairs Medical Center, Cleveland, Ohio

**Keywords:** Tigecycline, *Clostridium difficile*, ceftriaxone, piperacillin/tazobactam, linezolid, metabolomics, colonization resistance

## Abstract

**Background:**

The glycylcycline antibiotic tigecycline may have a relatively low propensity to promote *Clostridium difficile* infection in part because it causes less disruption of the indigenous intestinal microbiota than other broad-spectrum antibiotics. We used a mouse model to compare the effects of tigecycline versus other commonly used antibiotics on colonization resistance to *C. difficile* and on the metabolic functions of the intestinal microbiota.

**Methods:**

To assess *in vivo* colonization resistance to *C. difficile*, mice were challenged with oral *C. difficile* spores 1, 7, or 12 days after completion of 3 days of treatment with subcutaneous saline, tigecycline, ceftriaxone, piperacillin-tazobactam, or linezolid. Levels of bacterial metabolites in fecal specimens of mice treated with the same antibiotics were analyzed using non-targeted metabolic profiling by gas chromatograph (GC)/mass spectrometry (MS) and ultra-high performance liquid chromatography-tandem MS (UPLC-MS/MS).

**Results:**

All of the antibiotics disrupted colonization resistance to *C. difficile* when challenge occurred 2 days after treatment. Only piperacillin/tazobactam mice had disturbed colonization resistance at 7 days after treatment. All of the antibiotics altered fecal metabolites in comparison to controls, but tigecycline caused significantly less alteration than the other antibiotics, including less suppression of multiple amino acids, bile acids, and lipid metabolites.

**Conclusions:**

Tigecycline, linezolid, and ceftriaxone caused transient disruption of colonization resistance to *C. difficile*, whereas piperacillin/tazobactam caused disruption that persisted for 7 days post-treatment. Tigecycline caused less profound alteration of fecal bacterial metabolites than the other antibiotics, suggesting that the relatively short period of disruption of colonization resistance might be related in part to reduced alteration of the metabolic functions of the micro-biota.

## INTRODUCTION

Antimicrobial therapy plays a central role in the pathogenesis of *Clostridium difficile* infection (CDI). Nearly all antibiotics have been associated with the development of CDI, but clindamycin, cephalosporins, penicillins, and fluoroquinolones are generally considered the agents that pose the greatest risk [1]. Several recent studies suggest that antibiotics with inhibitory activity against *C. difficile* (eg, piperacillin/tazobactam) might be less likely than non-inhibitory antibiotics to promote CDI [1–3]. However, the potential to prevent CDI through selective prescription of agents with inhibitory activity against *C. difficile* may be limited if these agents also disrupt the indigenous microbiota of the colon. For example, piperacillin/tazobactam inhibits colonization by *C. difficile* in mice during treatment, but promotes growth and toxin production if exposure occurs during the period of recovery of the anaerobic microbiota [1].

Tigecycline is a broad-spectrum glycylcycline antibiotic that is administered intravenously. For several reasons, it is plausible that tigecycline might have a relatively low propensity to promote CDI in comparison to other broad-spectrum antibiotics. First, tigecycline has potent activity against *C. difficile* and is excreted in significant concentrations in bile (median fecal concentration in human volunteers, 5.6 mg/kg on day 8 of administration) [4]. In a human gut model, tigecycline inhibited growth and toxin production by *C. difficile* [5]. In mice, tigecycline inhibited establishment of colonization, but did not reduce concentrations of *C. difficile* once high-density colonization was established [6–7]. Second, there is some evidence that tigecycline may cause relatively limited disruption of the indigenous microbiota of the colon, with sparing of *Bacteroides* spp. [4,6]. However, in some studies, tigecycline has caused significant alteration of the microbiota, including *Bacteroides* spp. [5, 7–8]. Finally, tigecycline inhibits protein synthesis and has been shown to inhibit *C. difficile* toxin production and sporulation in mice [8].

In addition to providing colonization resistance, the intestinal microbiota provide a number of metabolic functions that benefit the host [9–10]. In mice, we demonstrated that recovery of colonization resistance to *C. difficile* after clindamycin or piperacillin/tazobactam treatment coincided with restoration of pretreatment levels of several fecal bacterial metabolites [9]. Here we used the same mouse model to test the hypothesis that tigecycline causes less alteration of fecal bacterial metabolites and colonization resistance to *C. difficile* compared to other broad-spectrum antibiotics. The impact of the antibiotics on levels of bacterial metabolites in fecal specimens was examined using non-targeted metabolic profiling by gas chromatograph (GC)/mass spectrometry (MS) and ultra-high performance liquid chromatography-tandem MS (UPLC-MS/MS).

## METHODS

### *C. difficile* strain

The *C. difficile* strain studied was ATCC 43593, a nontoxigenic strain from the American Type Culture Collection (ATCC). We prepared spores as previously described [11], growing *C. difficile* on Duncan and Strong agar. Harvested using sterile swabs, the spores underwent alcohol shock followed by sonication to break up vegetative material. The minimum inhibitory concentrations (MICs) of tigecycline, piperacillin-tazobactam, linezolid, and ceftriaxone were < 0.012 μg/mL, < 1 μg/mL, < 0.05 μg/mL, and > 256 μg/mL, respectively, as determined by broth dilution.

### Mouse model of in vivo colonization resistance to *C. difficile*

The Animal Care Committee of the Cleveland Veterans Affairs Medical Center approved the experimental protocol. The experimentation was performed in compliance with the U.S. Department of Health and Human Services Guide for the Care and Use of Laboratory Animals. Female CF-1 mice weighing 25 to 30 g (Harlan Sprague-Dawley, Indianapolis, IN) were housed in individual cages. Mice received daily subcutaneous injections (0.2 mL total volume) of saline, tigecycline (0.05 mg/day), ceftriaxone (1 mg/day), piperacillin-tazobactam (8 mg/day), or linezolid (0.6 mg/day) for 3 days. The antibiotic doses were equal to the usual human doses administered over a 24-hour period (milligrams of antibiotic per gram of body weight). At 2, 7, or 9 days after the final antibiotic dose, mice were administered 10^3^ CFU *C. difficile* spores (ATCC 43593) in 0.4 mL of sterile water by oral gavage. Mice (6 per group) received daily treatment with the antibiotics as noted above and fecal specimens were collected prior to treatment on day 3 of treatment, and at 2- to 5-day intervals after the final antibiotic dose, for analysis of the intestinal microbiota and stool metabolites.

### Impact of antibiotic treatment on enterococci and facultative Gram-negative bacilli

To quantify the burden of *C. difficile*, fresh stool samples were collected on days 1, 3, and 7 after the administration of spores. Samples were transferred to an anaerobic chamber (Coy Laboratories, Grass Lake, Michigan), emulsified 1:10 (weight/volume) in pre-reduced phosphate-buffered saline (PBS), serially diluted, and plated on pre-reduced cycloserine-cefoxitin-brucella agar containing 0.1% taurocholic acid and 5 mg/mL lysozyme (*C. difficile* Brucella Agar [CDBA]) [11]. Fresh stool specimens were used for quantitative culture of enterococci and facultative Gram-negative bacilli. The specimens were emulsified in 5-fold (weight/volume) pre-reduced phosphate-buffered saline. Serially diluted aliquots were inoculated onto selective agar. Plates were incubated at 37°C for 48 hours and CFU per gram of stool were calculated.

### Fecal metabolite analysis

Analysis of metabolic compounds in fecal specimens was conducted by Metabolon, Inc. (Durham, NC) using the methods described previously [9, 12–13]. Fecal samples underwent a methanol extraction, and the resulting extract was divided into fractions for analysis using GC/ MS, UPLC-MS/MS (positive mode), and UPLC-MS/MS (negative mode). The UPLC-MS/MS platform utilized an Acquity UPLC (Waters, Milford, CA) with a Waters UPLC BEH C18-column 2.1×100 mm, 1.7 μm and a ThermoFisher LTQ mass spectrometer, which included an electrospray ionization source and a linear ion-trap mass analyzer. The instrumentation was set to monitor for positive ions in acidic extracts or negative ions in basic extracts through independent injections. The instrument was set to scan 99–1000 m/z and alternated between MS and MS/MS scans. Samples destined for analysis by GC-MS were dried under vacuum desiccation for a minimum of 18 hours prior to being derivatized using bis(trimethylsilyl) trifluoroacetamide. Derivatized samples were separated on a 5% phenyldimethyl silicone column with helium as carrier gas and a temperature ramp from 60° to 340°C within a 17-min period. All samples were analyzed on a Thermo-Finnigan Trace DSQ fast-scanning single-quadrupole MS operated at unit mass resolving power with electron impact ionization and a 50–750 atomic mass unit scan range.

Metabolites were identified by automated comparison of the ion features in the experimental samples to a reference library of chemical standard entries that included retention time, molecular weight (m/z), preferred adducts, and in-source fragments as well as associated MS spectra, and were curated by visual inspection for quality control using software developed at Metabolon. Identification of known chemical entities was based on comparison to metabolomic library entries of more than 2,400 purified standards. An additional 5,300 mass spectral entries have been created for structurally unnamed biochemicals, which have been identified by virtue of their recurrent nature (both chromatographic and mass spectral). Peaks were quantified using area under the curve. Raw area counts for each metabolite in each sample were normalized to correct for variation resulting from instrument inter-day tuning differences by the median value for each run-day; therefore the medians were set to 1.0 for each run. This preserved variation between samples but allowed metabolites of widely different raw peak areas to be compared on a similar graphical scale. Missing values were imputed with the observed minimum after normalization. Biochemicals were mapped to pathways using the Kyoto Encyclopedia of Genes and Genomes (KEGG) database [14] or the Human Metabolome Database [15].

### Data Analysis

Analysis of variance (ANOVA) with repeated measures was used to compare concentrations of microbiota species or groups of control versus antibiotic-treated mice. To account for multiple comparisons in each group we applied Bonferroni's correction and considered *P* < 0.005 to be statistically significant. For the metabolite analysis, ANOVA with repeated measures was performed after log transformation to identify compounds that differed significantly between control and antibiotic-treated groups. The fold change in the concentrations of the compounds determined to be of importance in distinguishing antibiotic-treated versus control mice in comparison to the baseline levels was graphed over time. For purposes of analysis, we considered compounds with a 10-fold or greater increase or decrease in antibiotic-treated mice versus control mice to be meaningful. Data analyses were performed using R software (version 2.10.1).

## RESULTS

### Mouse model of in vivo colonization resistance to *C. difficile*

**Figure 1.**
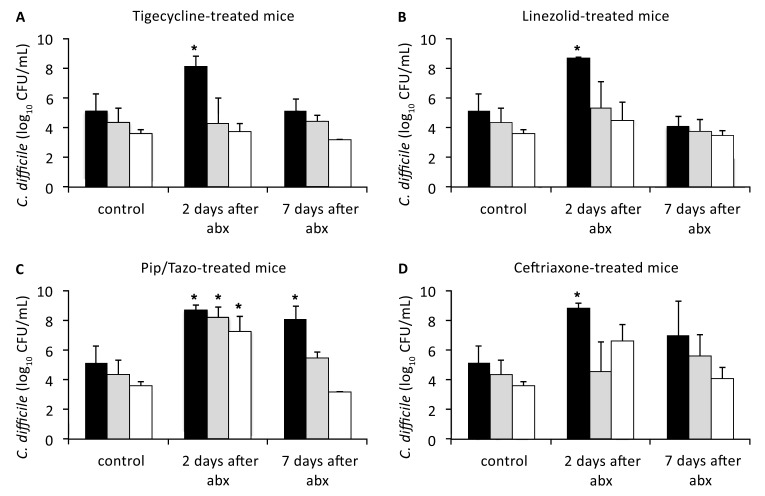
Timing of recovery of *in vivo* colonization resistance following antibiotic treatment. Mice (3 per group at each time point) were challenged with 10^4^ colony-forming units of non-toxigenic *Clostridium difficile* spores by orogastric gavage 2 or 7 days following treatment with 3 days of daily subcutaneous tigecycline (A), linezolid (B), piperacillin/tazobactam (C), or ceftriaxone (D). Concentrations of *C. difficile* in feces were measured by quantitative cultures 1 (black bars), 3 (grey bars), or 7 (white bars) days following challenge. *P < 0.005 indicates comparison of antibiotic-treated mice to control animals at the corresponding time point after *C. difficile* challenge. Error bars represent standard error. Results are shown for 1 of 2 duplicate experiments. Abx, antibiotics, Pip/Tazo, piperacillin/tazobactam.

Figure 1 shows the effect of antibiotic treatment on the establishment of colonization with *C. difficile* when mice were challenged 2 or 7 days after the last antibiotic dose. In comparison to saline controls, mice in all of the antibiotic groups initially developed high concentrations of *C. difficile* in stool (*P* < 0.005) when challenged 2 days after the last antibiotic dose. Mice treated with piperacillin/tazobactam and challenged 7 days after the last antibiotic dose also developed significant overgrowth of *C. difficile* in comparison to controls (*P* < 0.001), whereas those treated with tigecycline, linezolid, or ceftriaxone did not. At 9 days after the last antibiotic dose, none of the treatment groups developed overgrowth of *C. difficile* after challenge by oral gavage (data not shown).

### Effect of treatment on enterococci and facultative Gram-negative bacilli by culture

**Figure 2.**
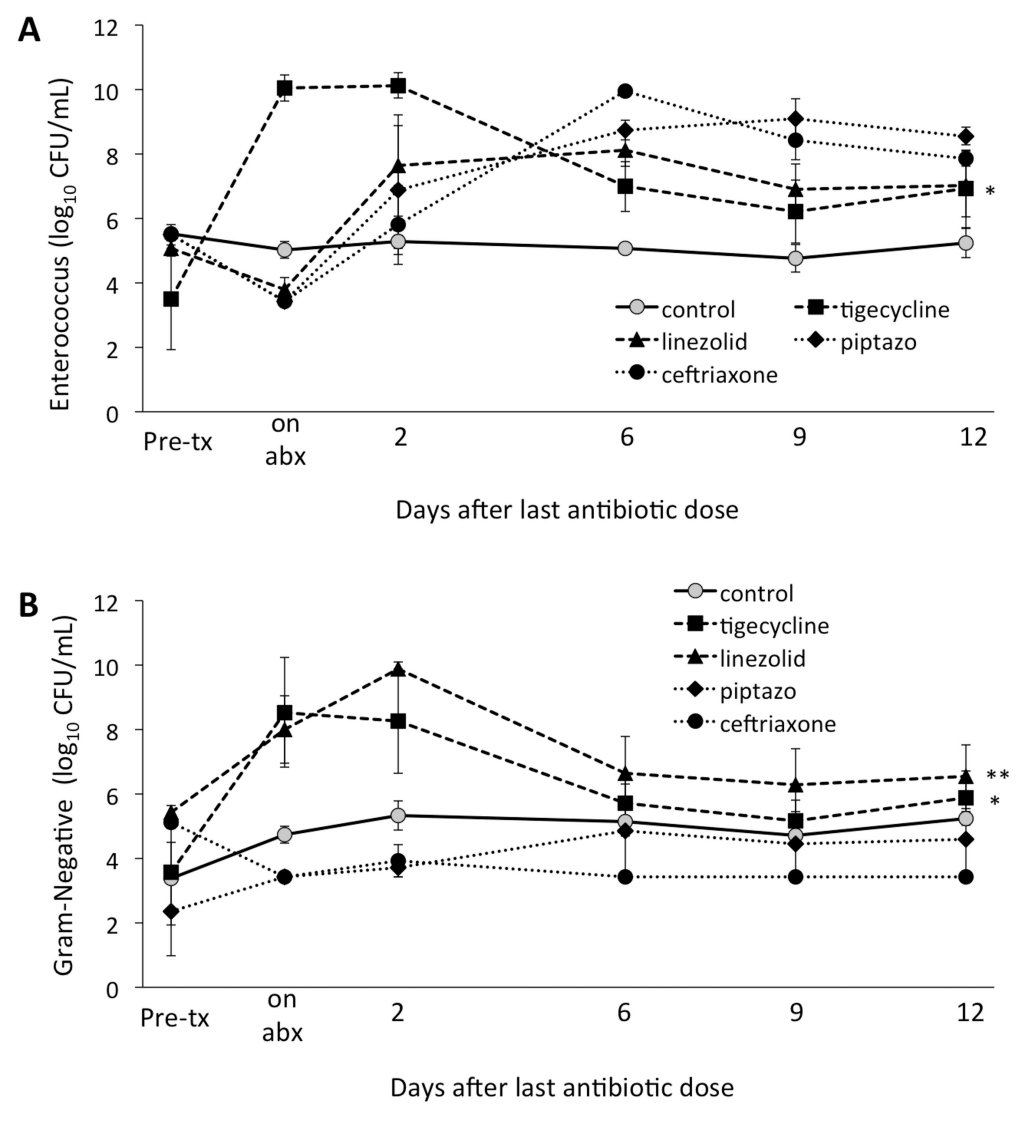
Timing of recovery of enterococci (A) and facultative Gram-negative bacilli (B) by quantitative culture following antibiotic treatment. Mice (6 per group) received subcutaneous antibiotics or normal saline for 3 days. Quantitative cultures were used to measure total enterococci or facultative Gram-negative bacilli in fecal specimens collected either before treatment, during or following treatment. In both panels, * indicates *P* < 0.05 for tigecycline-treated animals compared to control animals over the course of the experiment, specifically from antibiotic exposure through day 12 after the last antibiotic dose. In panel B, ** indicates *P* < 0.005 for linezolid-treated animals compared to control animals, also over the course of the experiment. Error bars represent standard error. Abx, antibiotics; pip/tazo, piperacillin/tazobactam.

As shown in Figure 2A, in comparison to controls, tigecycline promoted overgrowth of enterococci during treatment, with recovery to levels not significantly different from controls by 6 days after the last antibiotic dose. In comparison to controls, linezolid, piperacillin/tazobactam, and ceftriaxone resulted in significantly reduced levels of enterococci during treatment (*P* < 0.01), with a subsequent rebound after discontinuation of treatment to concentrations significantly higher than controls (*P* < 0.01). As shown in Figure 2B, piperacillin/tazobactam and ceftriaxone suppressed levels of facultative Gram-negative bacilli during treatment (*P* < 0.001), whereas tigecycline and linezolid promoted overgrowth (*P* < 0.01).

### Fecal metabolite analysis

**Figure 3.**
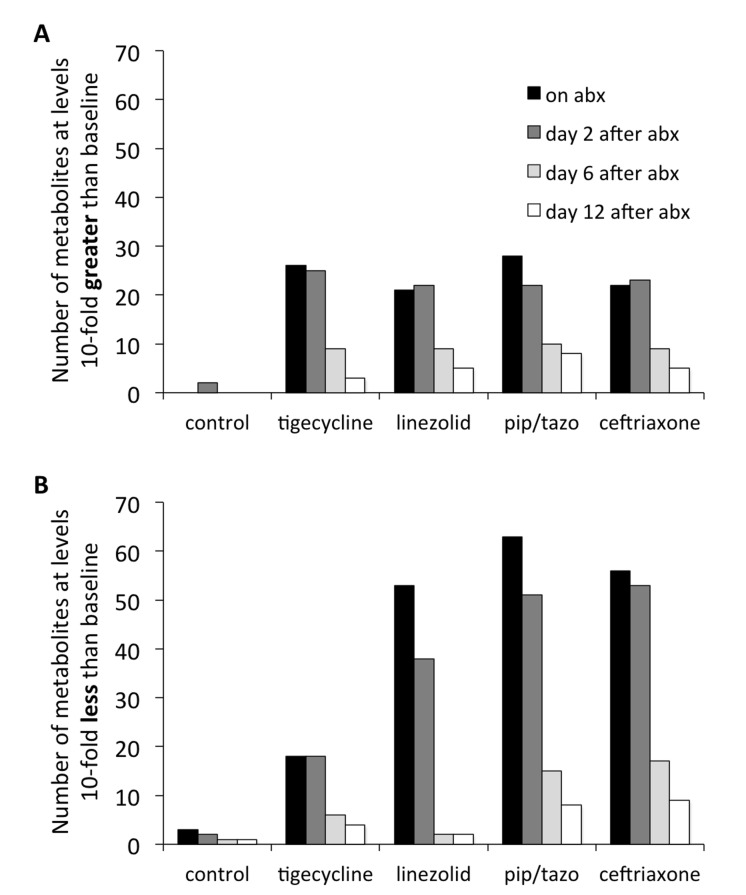
Impact of antibiotic treatment on fecal metabolites. Numbers of fecal metabolites with a 10-fold increase (A) or decrease (B) compared to control animals during antibiotic treatment (black bars) and at 2 days (dark grey bars), 6 days (light grey bars), and 12 days (white bars) following antibiotics. Abx, antibiotics, pip/tazo, piperacillin/tazobactam.

Five-hundred fifteen compounds were identified in the fecal samples. Figure 3 shows the number of metabolites exhibiting a 10-fold or greater increase or decrease in concentration in comparison to baseline. Each of the antibiotics resulted in 10-fold increases in 20 or more compounds during treatment (day 3) and on day 2 after treatment, but fewer than 10 compounds remained elevated 10-fold by day 12 after treatment. Piperacillin/tazobactam, ceftriaxone, and linezolid treatment resulted in transient 10-fold decreases in concentrations of 50 or more compounds, whereas tigecycline caused such changes in only 18 compounds.

Table 1 provides an overview of the metabolic super-pathways in which 10-fold or greater alterations in fecal metabolites occurred in mice treated with the 3 antibiotics causing the most profound alteration (ie, piperacillin/tazobactam, ceftriaxone, and linezolid) or in all 4 antibiotics. The metabolic sub-pathways and names of the metabolites are shown in Table 2. In contrast to the other antibiotics, tigecycline caused less suppression of a wide range of amino acid, bile acid, and lipid metabolites. Figure 4 provides examples of the impact of the antibiotics on scaled intensity of specific compounds. Table S1 shows the metabolites analyzed and the effect of antibiotic treatment expressed as a ratio of the metabolites detected in the fecal material of treated animals versus control animals at each time point.

**Table 1.** SuperPathways of Fecal Metabolites That Show a 10-fold Change vs. Baseline^a^

Metabolic SuperPathway^b^	10-fold decrease			10-fold increase		
	3 abx^c^	4 abx	total	3abx^c^	4 abx	total
Amino Acid	7	1	8			
Carbohydrate	3	2	5	2	2	4
Lipids, Bile Acids	6	1	7			
Lipids, other	5	0	5			
Nucleotides	1	0	1			
Peptides				1	1	2
Xenobiotics	3	0	3	3	3	5

a Considers 10-fold changes during or 2 days following completion of antibiotics but not at 6 or 12 days after completion of antibiotics.

b Metabolic sub-pathways and biochemical names of metabolites are in Table 2.

c abx, antibiotics; linezolid, piperacillin/tazobactam, and ceftriaxone

**Table 2.** Metabolic Subpathways and Biochemicals of Fecal Metabolites That Show a 10-fold Change vs. Baseline^a^

Super-Pathway	Sub-Pathway	Biochemical Name	Tigecycline	Linezolid	Piperacillin/ tazobactam	Ceftriaxone
Amino acid	Glycine, serine and threonine metabolism	sarcosine (N-Methylglycine)		1^b^	1	1
Amino acid	Phenylalanine and tyrosine metabolism	2-(4-hydroxyphenyl)propionate		1	1	1
Amino acid	Phenylalanine and tyrosine metabolism	4-hydroxyphenylacetate		1	1	1
Amino acid	Phenylalanine and tyrosine metabolism	phenylacetate		1	1	1
Amino acid	Urea cycle; arginine-, proline-, metabolism	2-aminopentanoate		1	1	1
Amino acid	Urea cycle; arginine and proline metabolism	5-aminovalerate	1	1	1	1
Amino acid	Valine, leucine, and isoleucine metabolism	3-methyl-2-oxobutyrate		1	1	1
Amino acid	Valine, leucine, and isoleucine metabolism	4-methyl-2-oxopentanoate		1	1	1
Carbohydrate	Amino-sugars metabolism	N-acetylglucosamine		1	1	1
Carbohydrate	Amino-sugars metabolism	N-acetylneuraminate	1	1	1	1
Carbohydrate	Fructose, mannose, galactose, starch, and sucrose metabolism	galactinol		10	10	10
Carbohydrate	Fructose, man-nose, galactose, starch, and sucrose metabolism	mannitol		10	10	10
Carbohydrate	Fructose, man-nose, galactose, starch, and sucrose metabolism	N-acetylmuramate	1	1	1	1
Carbohydrate	Fructose, man-nose, galactose, starch, and sucrose metabolism	raffinose	10	10	10	10
Carbohydrate	Fructose, man-nose, galactose, starch, and sucrose metabolism	sorbitol	10	10	10	10
Carbohydrate	Nucleotide sugars, pentose metabolism	arabinose		1	1	1
Carbohydrate	Nucleotide sugars, pentose metabolism	xylose		1	1	1
Lipid	Bile acid metabolism	12-dehydrocholate	1	1	1	1
Lipid	Bile acid metabolism	3-dehydrocholate		1	1	1
Lipid	Bile acid metabolism	7,12-diketolithocholate		1	1	1
Lipid	Bile acid metabolism	alpha-muricholate		1	1	1
Lipid	Bile acid metabolism	beta-muricholate		1	1	1
Lipid	Bile acid metabolism	cholate		1	1	1
Lipid	Bile acid metabolism	lithocholate [6-oxo or 7-keto]		1	1	1
Lipid	Fatty acid metabolism	isovalerate		1	1	1
Lipid	Fatty acid, branched	13-methylmyristic acid		1	1	1
Lipid	Fatty acid, dicarboxylate	sebacate (decanedioate)		1	1	1
Lipid	Long-chain fatty acid	conjugated linoleate (18:2n7; 9Z,11E)		1	1	1
Lipid	Sphingolipid	3-ketosphinganine		1	1	1
Nucleotide	Purine metabolism, guanine-containing	guanine		1	1	1
Peptide	Dipeptide	pyroglutamylvaline		10	10	10
Peptide	gamma-glutamyl	gamma-glutamylisoleucine	10	10	10	10
Xenobiotics	Food component/ plant	dihydroferulic acid		1	1	1
Xenobiotics	Food component/ plant	ferulate		1	1	1
Xenobiotics	Food component/ plant	Isobar: dihydrocaffeate, 3,4-dihy-droxycinnamate		1	1	1
Xenobiotics	Food component/ plant	melezitose		10	10	10
Xenobiotics	Food component/ plant	shikimate	10	10	10	10
Xenobiotics	Food component/ plant	soyasaponin I	10	10	10	10
Xenobiotics	Food component/ plant	soyasaponin II	10	10	10	10
Xenobiotics	Sugar, sugar substitute, starch	1-kestose		10	10	10

a Considers 10-fold changes during or 2 days following completion of antibiotics but not at 6 or 12 days after completion of antibiotics.

b 1 indicates a 10-fold decrease and 10 indicates a 10-fold increase compared to baseline values during or 2 days following antibiotics but not at 6 or 12 days following antibiotics.

**Figure 4A.**
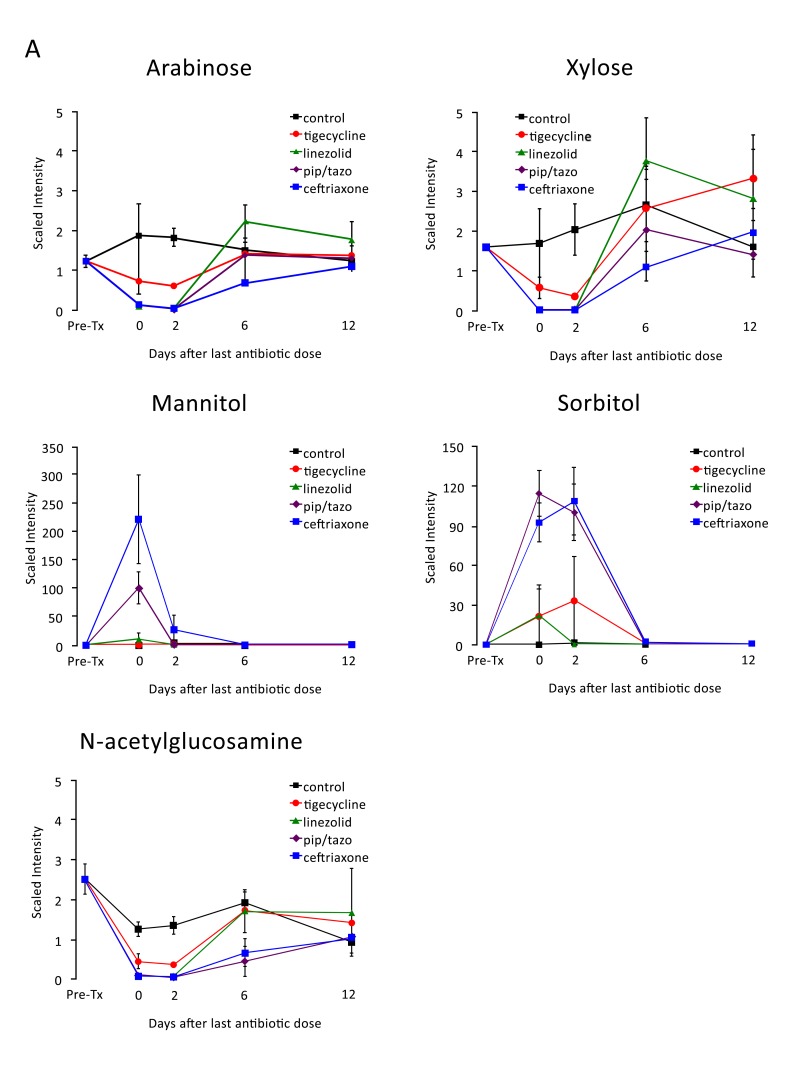


**Figure 4B-C.**
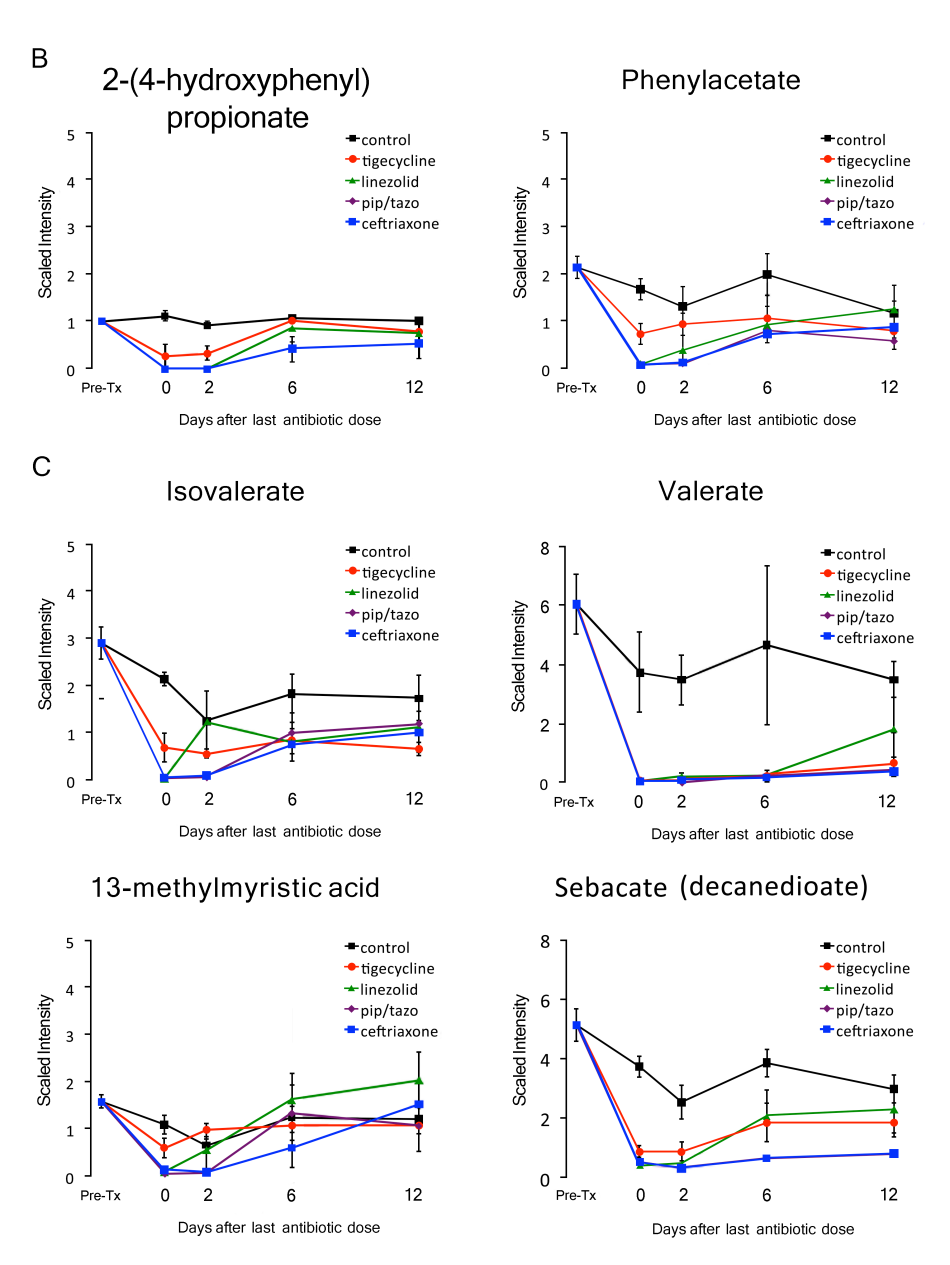


**Figure 4D.**
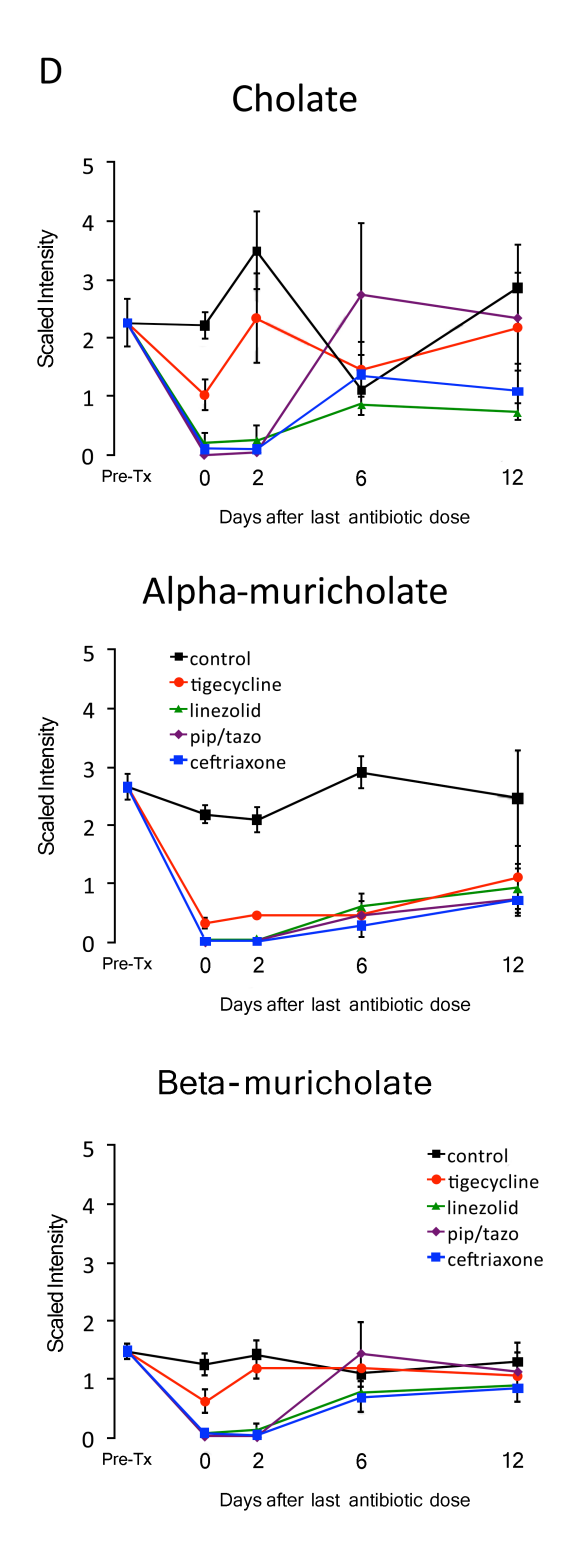
Changes in levels of fecal metabolites. Mice (4 per group at each time point) received subcutaneous antibiotics or saline for 3 days. Changes in the level of selected compounds from several different metabolic pathways: (A) carbohydrates; (B) phenylalanine and tyrosine; (C) fatty acids; and (D) bile acids. Error bars represent standard error. Pre-Tx, pre-antibiotic baseline. Pip/tazo, piperacillin/tazobactam.

## DISCUSSION

Broad-spectrum antibiotic therapy often results in unintended adverse consequences due to disruption of the indigenous microbiota of the host. In mice, we found that each of the antibiotics studied altered colonization resistance to *C. difficile* when challenged with spores 2 days post-treatment. However, only piperacillin/tazobactam caused disruption that persisted for 7 days post-treatment. Each of the antibiotics caused significant alteration of multiple fecal bacterial metabolites, but tigecycline caused less profound alteration than the other antibiotics. Thus, the relatively short period of disruption of colonization resistance by tigecycline might be related in part to the fact that this agent causes less alteration of the microbiota.

Our finding that antibiotic treatment dramatically alters fecal metabolites is consistent with other recent studies [9, 16–21]. In several studies in rodents, antibiotic treatment has been shown to reduce fecal excretion of amino acids and/or short chain fatty acids (SCFAs) [9, 16–19]. Theriot et al. reported that treatment of mice with the broad-spectrum antibiotic cefoperazone resulted in elevation of sugar alcohols and primary bile acids, and decreases in secondary bile acids and SCFAs [20]. Moreover, *C. difficile* was able to exploit these metabolic changes to colonize the intestinal tract, including use of the primary bile acid taurocholate for germination and sugar alcohols and other carbon sources for growth [20]. Finally, we previously reported that clindamycin or piperacillin/tazobactam treatment resulted in alteration of a wide range of fecal bacterial metabolites, including intermediates in carbohydrate or protein metabolism that increased (pentitols, gamma-glutamyl amino acids, and inositol metabolites) or decreased (pentoses, dipeptides) with treatment [9].

A number of the compounds that increased or decreased by 10-fold in concentration during antibiotic treatment demonstrated normalization within 6 to 12 days concurrent with recovery of colonization resistance (Figure 3). Thus, these compounds could potentially serve as biomarkers or mediators of colonization resistance. Some of these potential biomarkers were intermediates in carbohydrate or protein metabolism that increased during antibiotic treatment (eg, sugar alcohols, dipeptides, and gamma-glutamyl amino acids), presumably due to loss of metabolic digestion by the intestinal microbiota. Other potential biomarkers of colonization resistance decreased during antibiotic treatment and then showed evidence of recovery (eg, alpha-muricholate, lithocholate, beta-muricholate, N-acetylglucosamine, N-acetylneuraminate, isovalerate, and valerate). Several studies have suggested that secondary bile salts such as lithocholate may play a role in colonization resistance to *C. difficile* through inhibition of germination and outgrowth [22–23].

Our study has some limitations. The *in vivo* colonization resistance assessment included only one strain of *C. difficile* that was non-toxigenic. However, we have previously demonstrated that non-toxigenic *C. difficile* strains colonize mice similarly to toxigenic strains [9]. Our model did not provide an assessment of prolonged or repeated courses of broad-spectrum antibiotics that might cause more prolonged disruption of the microbiota. We did not include an analysis of the anaerobic microbiota in the current study. However, we have previously demonstrated that concentrations of *Bacteroides* spp. are not reduced in this mouse model [9], consistent with a previous study of healthy human volunteers [4]. Given the degree of functional redundancy of the intestinal microbiota, it is likely that multiple families of bacteria may be able to carry out the metabolic conversions required to produce the metabolites identified here. Tigecycline promoted overgrowth of enterococci and facultative Gram-negative bacilli in stool despite the fact that it has activity against many strains of these organisms. This observation may be due to the emergence of resistant strains of these types of bacteria, but we did not include an evaluation for emergence of resistant organisms in our study. Others have demonstrated that tigecycline treatment may suppress susceptible *Escherichia coli* while promoting overgrowth of total facultative Gram-negative bacilli due to the emergence of tigecycline-resistant strains (eg, *Klebsiella* and *Enterobacter* spp.) [4, 7].

## SUPPLEMENTARY MATERIALS

Supplementary Table 1 can be downloaded here in Excel format.
